# Screening tools for evaluation of depression in Chronic Obstructive Pulmonary Disease (COPD). A systematic review

**DOI:** 10.1080/20018525.2017.1332931

**Published:** 2017-06-12

**Authors:** K. Bock, E. Bendstrup, O. Hilberg, A. Løkke

**Affiliations:** ^a^ Department of Respiratory Diseases and Allergy, Aarhus University Hospital, Aarhus C, Denmark

**Keywords:** COPD, depression, anxiety, comorbidity, mortality, quality of life, screening instruments

## Abstract

**Background**: Anxiety and depression are common comorbid disorders in patients with chronic obstructive pulmonary disease (COPD), though estimates of their prevalence vary considerably. Depressive symptoms/depression are important comorbidities in COPD and an increasing interest is shown to these disorders. Depression may lead to reduced quality of life and increased morbidity and mortality. These statements underline the importance of implementing the use of screening instruments for depressive symptoms in a clinical setting.

This systematic review evaluates four commonly used screening tools for depression in COPD. Furthermore we assess the prevalence of depression in COPD in the evaluated studies.

**Design**: A literature search identified studies dealing with screening for depression in patients with COPD. We focused on the instruments: Beck Depression Inventory, Geriatric depression scale, Centre for Epidemiological Studies scale on Depression and Hospital and Anxiety Depression Scale.

**Results**: Overall prevalence of depression was 30%. Demographic variations and severity of COPD influenced prevalence. The inter-prevalence of the four screening tools was consistent. We found a low variation between studies using the same tool. Few studies used control groups or compared the screening tool to a psychiatrist evaluation.

**Conclusions**: This article calls for further investigation of the association between COPD and depressive symptoms. The subject is highly relevant for everyday life of patients with COPD and attention needs to be drawn to this issue in both an out- and in-patients.

## Introduction

Chronic obstructive pulmonary disease (COPD) is an increasing global health problem. By 2020, the World Health Organization (WHO) estimates COPD to be the fifth major cause of disability.[1] and by 2030 the third leading cause of death.[2] There is growing concern regarding the global burden of depression also affecting patients with COPD. WHO estimates that 350 million people are affected by depressive disorders and that depression is the third leading cause of disability.

Living with COPD means living with cough, dyspnoea and exertional impairment. Patients experience reduced daily activity and physical disability and impaired sleep quality. Dyspnoea frequently forces patients to stay at home leading to decreased quality of life, lack of social interaction and ultimately social isolation.

There is growing concern that depression and/or depressive symptoms are major factors influencing the quality of life and overall survival for COPD patients.[3,4] It has also been pointed out that functional impairment causes depression and that this depression is often more severe and leading to a high frequency of medical contacts.[5]

Therefore, it is of increasing interest to treat both mental and physical comorbidities to improve quality of life and prognosis for patients with COPD.[6]

Assessment of depression in patients with COPD is difficult. The gold standard of diagnosing depression is by an interview with a psychiatrist but from a clinical point of view self-rating instruments or screening instruments that can be used and evaluated by clinicians are highly appreciated to make the initial evaluation of the patients. However, several important aspects of COPD interfere with characteristics seen in depression such as fatigue, weight loss, sleep disturbance and sadness among others, thus making interpretation difficult. There is an ongoing concern for the underdiagnosis and thereby possible undertreatment of depression in COPD patients.

Through a systematic review of existing literature, this article presents accurate knowledge of the prevalence of depressive symptoms in COPD. Furthermore, it describes and evaluates the four most common and best validated screening instruments available to assess depressive symptoms in COPD.

## Method

**Figure 1.**
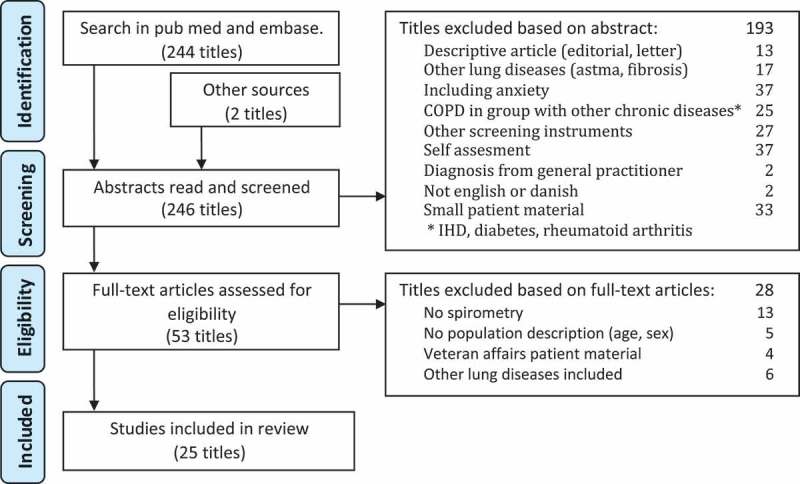
The search strategy and results.

A literature search was performed in the databases PubMed and Embase. Medical Subject Headings (MeSH-terms) were searched for using MeSH database. The search word ‘depression’, MeSH [depression] and [depressive disorder] was chosen. Search in MeSH database for ‘COPD’, MeSH [Pulmonary Disease, Chronic Obstructive] was chosen. PubMed search: MeSH [Pulmonary Disease, Chronic Obstructive] AND MeSH [depression] OR MeSH [depressive disorder]. Criteria for inclusion were English language articles and available abstract. Furthermore, we performed snowball search. The search resulted in 246 articles (Figure 1).

Abstracts were read and relevant articles selected based on our inclusion and exclusion criterias. Afterwards eligible articles were read and data extraction performed for every article. The data were listed according to the 4 screening tools described in this review as illustrated in Table 1.

**Table 1.** Data from the studies included i review.

	Author	Design	Sample size	Age, Mean	Male (%)	FEV 1 (%) or GOLD-group	Prevalence of depressive symptoms
**GDS**	Lacasse, Canada; 2001 [11]	Cross sectional	109 (105 LTOT*)	71	58%	34%	75%
	Julian, United States; 2009 [12]	Cross sectional	188	66	40%	49%	25%
	Omachi, United States; 2009 [13]	Cross sectional	1202	58	63%	I-IV	26% (Gold I-II); 34% (Gold III-IV); control 5,6%
	Ng, China; 2009 [14]	Cross sectional	189	65 (55–74)	35%	II-IV	23%
	**Subtotal (weighted)**		**1688**	**60**	**57%**		**31%**
**BDI**	de Voogd, Holland; 2009 [15]	Cross sectional	121	61	65%	36%	20%
	Chavannes, Holland; 2005 [16]	Cross sectional	147	58	75%	63%	27%
	Fan, United states; 2007 [17]	Cross sectional	610	66	64%	27%	40%
	Wagena, The Netherlands; 2005 [18]	Cross sectional	118	58	55%	56%	29%
	Doyle, United states; 2013 [19]	Cross sectional	162	67	62%	47%	28%
	Papaioannou, Greece; 2013 [3]	Cross sectional	230	71	88%	52%	39%
	**Subtotal (weighted)**		**1388**	**65**	**68%**		**34%**
**CES-D**	van Manen, Holland; 2002 [20]	Cross sectional	162	67	70%	60 pt FEV1 < 50%; 102 pt FEV1 50–80%	25% (FEV1 < 50%;) 19% (FEV1 50–80%)
	Al Shair, England; 2009 [21]	Cross sectional	122	66	61%	52%	21%
	Coultas, United States; 2007 [22]	Cross sectional	207	69	45%	II-IV	60%
	Hanania, Multinational study; 2010 [23]	Cross sectional	2118	63	65%	48%	26%
	Stapleton, United States; 2003 [24]	Cross sectional	101 (LTOT)	67	77%	26%	45%
	Hayashi, Japan; 2011 [25]	Cross sectional	131	73	100%	51%	29%
	**Subtotal (weighted)**		**2841**	**64**	**66%**		**29%**
**HADS**	Hayashi, Japan; 2011 [25]	Cross sectional		73	100%	51%	40%
	de Voogd, Holland; 2009 [4]	Follow up	122	60	48%	41%	33%
	Bratås, Norway; 2010 [26]	Follow up	136	65	49%	I-IV (60% III-IV)	27%
	Cleland, United kingdom; 2007 [27]	Cross sectional	110	67	52%	I-IV	21%
	Funk, Austria; 2009 [28]	Cross sectional	122	65	55%	45%	52%
	Janssen, Holland; 2010 [29]	Cross sectional	701	63	60%	44%	27%
	Stoilkova, Holland; 2013 [30]	Cross sectional	303	62	53%	47%	29%
	Bhandari, United states; 2013 [31]	Follow up	366	69	51%	47%	17%
	Lou, China; 2012 [32]	Cross sectional	1100	62	75%	45%	35%
	Harrison, United kingdom; 2012 [33]	Cross sectional	518	69	57%	40%	17%
	**Subtotal (weighted)**		**3609**	**65**	**63%**		**28%**
	**Total (weighted)**		**9526**	**64**	**63%**		**30%**

*LTOT: Long term oxygen treatment; **GDS: Geriatric Depression Scale, *** BDI: Becks Depression Inventory, ^y^CES-D: Centre for Epidemiological Studies scale on Depression, ^Z^HADS: Hospital and Anxiety Depression Scale

We performed subtotal and total weighted means of the data.

### Criteria for inclusion

Studies had to include patients with COPD diagnosed by spirometry and had to meet the diagnostic criteria for COPD according to The Global Initiative for Chronic Obstructive Lung Disease (GOLD) guidelines.[7] More than one hundred of the excluded studies failed to meet these criteria mostly because of patient self-reported COPD-diagnosis, missing COPD-diagnosis or lack of spirometry result reflecting COPD severity.

Most existing studies on COPD and depression are epidemiological and prospective. We decided on an arbitrary lower limit of 100 patients as a minimum sample size to ensure a sample large enough to estimate a proper prevalence of depressive symptoms in the cohort.

Moreover smaller studies tended to evaluate a subgroup of patients such as patients known with psychiatric disorders and COPD adjacent to that. Other papers concentrated solely on COPD patients on long-term oxygen treatment and with very few participants.

Studies with patients originating from the veteran affairs health care services (former soldiers now being treated in a formal program) were excluded in order not to introduce bias as investigations have shown a higher prevalence of posttraumatic stress disorder and also of depressive symptoms among these patients.[8,9]

Using screening instruments as a tool to assess depressive symptoms was a mandatory criteria for inclusion. The following were included:

- Beck Depression Inventory (BDI)
- Geriatric depression scale (GDS)
- Centre for Epidemiological Studies scale on Depression (CES-D)
- Hospital and Anxiety Depression Scale (HADS)

The four screening instruments used for inclusion were selected primarily because they have all been validated in patients with somatic illness. Secondly, all four instruments are among the most commonly used in the literature. Furthermore, we wanted to evaluate the usefulness of validated screening instruments from a clinical point of view and give recommendations on how to approach depressive symptoms in COPD patients.

Studies using various other screening tools such as Zung, PHQ-2 (Patient Health Questionnaire), PHQ-9, BASDEC (Brief Assessment Schedule Depression Cards) and GHQ (General Health Questionnaire) were excluded since these are either poorly or not at all validated for this patient group or they are only used in a single or very few studies.[10]

Articles with depression diagnosed by the general practitioner or suspected depression diagnosed upon information of medication use were not included.

#### Screening instruments

In order to evaluate the four selected screening instruments and to address their strengths and limitations, we investigated:

1. Group of interest
2. Number of items – overall and with somatic symptoms
3. Sub-scales
4. Validation
5. Cut-off values

## Results

Results are listed according to the screening tool used in the studies.

Twenty-one studies originated from Europe and the United States, three from Asian countries and none from South America or Africa. There was one multicentre study.

Twenty-four studies were performed in a single country and were thus influenced by the culture and perception of mental mood disorders of that particular country.

Studies included both in- and out-patients. Patients were recruited from general practitioners as well as from medical hospital wards. Thus, both stable patients and patients with a recent COPD exacerbation were included. The timing of screening after hospital admission was not similar, but we were unable to account for this in our study.

### Geriatric Depression Scale (GDS)

1. The GDS was originally designed for use in elderly. It has been validated and is commonly used for different diseases such as COPD, IHD and diabetes mellitus. It is used for both in- and out-patients.
2. GDS 30 consists of 30 items. One item deals with somatic manifestations in terms of fatigue. Furthermore, GDS 15 is a 15-item questionnaire targeted at weakened patients with low energy.
3. GDS does not embrace symptoms of anxiety.
4. GDS has not been specifically validated in patients with COPD.
5. There are no absolute cut off values. GDS 30 typically uses scores of 10–11 for defining minor depressive symptoms. GDS 15 uses scores of 5–6.[34,35]

The four studies using the GDS as a screening tool for depression included 1688 patients with COPD; 57% were male patients and mean age was 60 years. All four GOLD groups of patients with COPD were represented. The overall percentage of patients with depressive symptoms was 31%.

Lacasse et al. [11] included older and more severely affected patients and found a higher percentage of patients with depressive symptoms (3 out of 4 patients) compared to the three other studies identifying one out of four patients with depressive symptoms.

### Beck Depression Inventory (BDI)

1. The BDI was developed in 1961 as a tool for clinicians to assess manifestations of depressive illness. It was adjusted in 1976 and again in 1996 where items less specific for depression such as weight loss, somatic preoccupation and inability to work were replaced with agitation, worthlessness and energy loss. It is now coherent with the DSM classification on the diagnosis of depression.
2. BDI is a self-reported inventory and consists of 21 items with possibility to score up to 70 points. Four items deal with somatic symptoms i.e. loss of appetite and sleep disturbances.
3. BDI has not been used as a screening tool for anxiety. Depression is divided into minor and major groups depending on total score.
4. The tool has been validated in groups with e.g. post-AMI, cancer and diabetes. It has not yet been validated in COPD. Caution should be taken in screening elderly people as there is a risk of either minimizing or over-reporting symptoms in this group.
5. A cut off of 13/14 for minor depressive symptoms is recommended by the author and is still accepted. The cut off value for patients with COPD still needs to be defined.[36–38]

1388 patients with COPD have been screened for depression using the BDI in six studies; 68% of the patients were male and mean age was 65 years.

Four of the studies included patients with moderate COPD and found a prevalence of depressive symptoms of 28% with the exception of the study from Papaioannou (39%). This study had more men included in the population and they were older compared with the other studies.

Two studies included patients with severe COPD; these studies found a higher prevalence of 37% with depressive symptoms.

### Centre for Epidemiological Studies scale on Depression (CES-D)

1. The CES-D is a self-report screening instrument made by L.S. Radloff in 1977 for assessment of depression in a general population.
2. CES-D is a 20-item phrased score with self-statements assessments. Items involve both somatic and social factors. Items are not fully congruent with DSM classification of depression.
3. No sub-scale on anxiety exists.
4. CES-D has not been validated in patients with COPD. There is growing concern on the validity of CES-D, as women tend to score high on this scale. The phrases used in the items seem to be more appealing to women e.g. ‘I had crying spells’. Also, persons with social difficulties tend to score high. Moreover, there are a higher number of somatic items than in other scales. Recent work suggests a modified 14-item scale adjusted to recent diagnostic criteria. This is not yet established.
5. No commonly used cut off values could be found.[39,40]

In the six studies using CES-D as a screening tool for depression, 2800 patients with COPD were included; 66% of the patients were males and mean age was 64 years.

Four studies primarily dealt with patients with moderate to severe COPD and found a 25% prevalence of depressive symptoms. Two studies (Coultas and Stapleton) included more patients with severe or very severe COPD (Stapleton et al. only include oxygen users) and found a prevalence of 50% for depressive symptoms.

### Hospital and Anxiety Depression Scale (HADS)

1. The HADS was constructed in 1983 by Zigmond and Snaith as a self-reported questionnaire ‘to measure depression and anxiety in patients in non-psychiatric hospitals.’ Today, it is used for both in- and out-patients.
2. HADS-D (depression) consists of seven items with a score from 0–21. To avoid overlap with chronic illness, there are no items concerning somatic symptoms.
3. HADS includes two subscales: a depression scale (HADS-D) and an anxiety scale (HADS-A).
4. HADS has been validated and is widely used in many different groups of patients, including patients with COPD.
5. Cut off values are recommended to be a composite value of both HADS-D and HADS-A subscales in order to include all cases of minor and major depression. A cut-off > 15 has been suggested.[41]

The ten studies using the HADS as a screening tool for depression includes 3609 patients with COPD; 63% of the patients were males and mean age was 65 years.

Two of the studies (Bratås and Cleland) look at patients with all stages of COPD and found a prevalence of depressive symptoms of 25%.

Bhandari and Harrison found a rather low prevalence of depressive symptoms (17%); the two studies do not differ on other measures.

The six remaining studies primarily deals with patients with moderate to severe COPD and found a prevalence of depressive symptoms ranging from 30 to 50%.

## Discussion

Earlier reviews on the association between depression and COPD have mostly been small studies including patients with different COPD severity (different FEV1% predicted, GOLD stage, use of LTOT), using different screening instruments and performed in different clinical settings. The strength of this review is the systematic way of dealing with and addressing the different depression scores in COPD.

The overall prevalence of clinically relevant depressive symptoms was found to be approximately three out of ten patients with COPD patients. This number is surprisingly stable with a very low variation between 28 and 34% among the four different screening tools evaluated in the current review (GDS, BDI, CES-D and HADS).

The inter-prevalence of each tool also displays great consistency despite minor variations. These variations are most likely due to differences in study populations. One study describes depression in a group with severe COPD using LTOT; this might reflect the higher prevalence.[11] Some studies recruit patients from rehabilitation; this group of patients is more likely to be socially and mentally well-functioning.[31,33] Bhandari and Harrison found a low prevalence of depressive symptoms. The two studies include patients participating in a rather comprehensive rehabilitation program. This might partly explain why patients able to participate in an extensive rehabilitation program might be less affected mentally.

Depression is a common comorbidity in COPD and this is corroborated in the present study reporting that patients with COPD display a higher prevalence of depressive symptoms than expected when compared to the background population. Earlier studies in the general population estimate an overall rate of depression that varies from 4 to 7%.[42] There are cultural and geographic differences with low estimates of depression i.e. in Japan and higher estimates in North Africa and Russia. In low income countries the estimates are to be interpreted with caution because of lack of well-organised healthcare system and registration. In Japan there is probably a great cultural aspect of psychiatric morbidity as the suicide rate in Japan is very high compared to other countries.[42]

Depression is widely accepted as a comorbid condition in COPD, but in every day clinical practice there is still a lack of attention to this issue. Papaioannou [3] and de Voogd [4] illustrate the relevance of dealing with depression in COPD, as there seems to be a tendency towards longer duration of hospitalisation in patients with a COPD exacerbation at the same time suffering from depression. Furthermore, a poorer survival in COPD is seen when combined with depression [3,4]. It is therefore of great relevance to implement screening for depression in both in- and out-patients.

In COPD patients we see an overlapping pattern of symptoms between anxiety and depression and the lung disease in itself. Willgoss et al. [43] investigate the load of anxiety disorders in COPD patients. They find comparable elevated prevalence of generalized anxiety disorders and additionally they draw the attention to social phobia as another significant psychiatric disorder.

The two psychiatric disorders often go hand in hand and to a COPD they cause further social isolation, weight loss and despair.

Only one in four described screening instruments assess the anxiety besides the depression. In a daily clinical setting the health care takers must draw attention to the disorders.

Different severity of COPD seems to be the most important single factor. There seems to be a correlation between COPD severity and the burden of depressive symptoms; Lou et al. [32] showed an overall prevalence of depressive symptoms of 35% but in patients with severe COPD, the prevalence was as high as 67% (p < 0.01, compared to mild and moderate COPD). This correlation is found in all of the four screening tools evaluated separately as well as when grouped together. Lacasse and Stapleton described depressive symptoms in patients with severe COPD using LTOT.[11,24] These studies showed rates of depressive symptoms as high as 75%.

The instruments we chose to evaluate are all used frequently in studies assessing depressive symptoms. None of the four scales seems to be superior to the others in terms of use and assessing depressive symptoms. Unfortunately, a direct comparison of the instruments is very difficult because of the diverse design of the studies. The study population comprises both in – and out-patients, smokers, former smokers and different educational levels – factors that all might influence the extent of depressive symptoms. The impact of each of these factors must be further evaluated in a clinical setting also considering important factors such as economy, license to use the instruments, accessibility and language. Not all instruments are translated to local languages which may necessitate translation to the patient by the clinical staff, validation and thereby elevating the costs of evaluating a patient with COPD.

The gold standard for diagnosis of mental depression is interview by a psychiatrist using ICD-10 criteria, but this is not possible in all patients with COPD patients and valid screening tools are thus needed.[44]

The tools evaluated in this article have all been validated for use in various chronic diseases. On behalf of the displayed findings we find reason to trust these four instruments to be able to identify patients at risk. Clinical staff should pay extra attention to these patients and consider the need for an evaluation by the general practitioner or a psychiatrist. This recommendation seems to be underlined by the following finding by Stage et al. [45] They evaluated the specific diagnoses of depression made by a psychiatrist against the Hamilton Depression Scale. This study showed coherence between the tool used and the psychiatric diagnosis. To our knowledge, there are no available studies evaluating the coherence between psychiatrist diagnosed depression and the findings of a validated screening tool for depressive symptoms.

Very few of the studies mentioned in this paper are designed as case/control studies. Omachi et al. [13] studied a group of patients with COPD and a control group matched on demographic characteristics. The level of disability due to COPD was measured (spirometry, dyspnoea and exercise capacity). There was a clear correlation between severity of COPD and the level of depressive symptoms compared to the control group.[13]

Al Shair [21] investigated depression among patients with COPD using the BODE-index (BMI, obstruction, dyspnoea and exercise capacity) as a marker of disease severity and also included a wide range of factors such as bioelectrical impedance and muscle wasting, 6 minute walk distance and St. Georges Respiratory Questionnaire. They found a clear association between depression and the BODE-index but not to FEV_1_ alone.

Zhang [46] could not demonstrate demographic and clinical varieties as moderators of the prevalence of depression. He found no correlation with age or lung function. Van Ede [47] found no evidence of a significant risk of depression in patients with COPD.

In conclusion, this review found that three out of ten patients with COPD have depressive symptoms and a possible depression when using any one of four validated instruments (GDS, BDI, CES-D and HADS).

The prevalence of depression identified by each tool displays great consistency and there seems to be a correlation between COPD severity and the burden of depressive symptoms. Only HADS is specifically validated in COPD.

The tools seem to identify patients with an increased risk of having a depression although well-designed studies investigating the coherence between psychiatrist diagnosed depression and the findings of a validated screening tool for depressive symptoms are lacking.
